# Developing interprofessional care plans in chronic care: a scoping review

**DOI:** 10.1186/s12875-016-0535-7

**Published:** 2016-09-21

**Authors:** Jerôme Jean Jacques van Dongen, Marloes Amantia van Bokhoven, Ramon Daniëls, Trudy van der Weijden, Wencke Wilhelmina Gerarda Petronella Emonts, Anna Beurskens

**Affiliations:** 1 Research Centre for Autonomy and Participation for People with Chronic Illnesses, Zuyd University of Applied Sciences, Nieuw Eyckholt 300, 6419 DJ Heerlen, The Netherlands; 2 Department of Family Medicine, Maastricht University, CAPHRI School for Public health and Primary Care, Maastricht, The Netherlands

**Keywords:** Systematic review, Interprofessional collaboration, Shared care plan, Goal setting, Scoping review, Chronic disease, Patient-centred practice

## Abstract

**Background:**

The number of people suffering from one or more chronic conditions is rising, resulting in an increase in patients with complex health care demands. Interprofessional collaboration and the use of shared care plans support the management of complex health care demands of patients with chronic illnesses. This study aims to get an overview of the scientific literature on developing interprofessional shared care plans.

**Methods:**

We conducted a scoping review of the scientific literature regarding the development of interprofessional shared care plans. A systematic database search resulted in 45 articles being included, 5 of which were empirical studies concentrating purely on the care plan. Findings were synthesised using directed content analysis.

**Results:**

This review revealed three themes. The first theme was the format of the shared care plan, with the following elements: patient’s current state; goals and concerns; actions and interventions; and evaluation. The second theme concerned the development of shared care plans, and can be categorised as interpersonal, organisational and patient-related factors. The third theme covered tools, whose main function is to support professionals in sharing patient information without personal contact. Such tools relate to documentation of and communication about patient information.

**Conclusion:**

Care plan development is not a free-standing concept, but should be seen as the result of an underlying process of interprofessional collaboration between team members, including the patient. To integrate the patients’ perspectives into the care plans, their needs and values need careful consideration. This review indicates a need for new empirical studies examining the development and use of shared care plans and evaluating their effects.

**Electronic supplementary material:**

The online version of this article (doi:10.1186/s12875-016-0535-7) contains supplementary material, which is available to authorized users.

## Background

As the average age in European countries rises, so does the number of people suffering from chronic diseases such as diabetes [1]. Depending on their age, 30–80 % of these patients with a chronic disease are confronted with multiple chronic conditions [2, 3].

Suffering from chronic conditions leads to considerable deterioration of functioning and increased care demands [4]. Chronically ill patients visit 4 − 9 different health care professionals regularly [5]. In order to keep rising health care costs under control, governments aim to shift the treatment of chronic patients from hospital care to primary care [6, 7]. The primary care setting will therefore be confronted with a substantial increase in workload, especially regarding patients with complex problems [5]. Accordingly, there is a need for effective and efficient interprofessional collaboration in chronic care, especially in the primary care setting [8]. Interprofessional collaboration can have positive effects on health care processes and outcomes [9]. In addition, interprofessional collaboration seems to be a prerequisite to facilitate a shift from disease-oriented to patient-oriented care [10].

Health care professionals are used to developing their own discipline-specific care plans. However, given the increasing complexity of care for people with multiple chronic conditions, it seems meaningful to synchronise these discipline-specific care plans into one interprofessional shared care plan. Interprofessional collaboration appears to be positively affected by the use of shared care plans [11–13]. Based on the literature, we define a shared care plan as a collaborative and shared document that involves a joint input from an interprofessional team of professionals [14], summarising the patient’s current and preferred situation, as well as personal goals and actions [15]. Developing shared care plans can be perceived as a means to improve the communication, coordination and synchronisation of care across health care professionals from a diversity of disciplines, resulting in more complete care plans [16]. In addition, the shared care plan should highlight the process of care, rather than being solely a chronically arranged list of interventions or tasks [17].

Although the use of shared care plans is recommended in various guidelines for chronic diseases, they have not been implemented on a large scale [15, 18]. Furthermore, there seem to be differences in both the content and structure of these care plans, and to date, they have rarely been patient-centred [18]. Various interrelated factors (both barriers and facilitators) influence the development of these care plans. Factors that have been mentioned as possible causes obstructing the development of such plans include poor coordination of care and lack of time in consultations [15]. San Martin-Rodriguez and colleagues [19] divided these factors into interactional determinants (processes related to interpersonal relationships), organisational determinants (aspects of the organisation), and systemic determinants (external factors) [19]. The development of tools and the use of health information technology have been acknowledged as possible strategies supporting the development of shared care plans.

Supporting implementation in practice would benefit from an overview of the scientific knowledge regarding the development process of interprofessional shared care plans. However, to our knowledge no overviews are available for this specific area. We therefore conducted a scoping review to explore the scientific literature on developing interprofessional shared care plans.

## Methods

### Study design

We explored the literature using a scoping review. We chose this approach, described by Arksey and O’Malley, because the area is complex and has not yet been reviewed comprehensively [20, 21]. In a scoping review, the inclusion of articles is merely based on the relevance of the studies, rather than on methodological quality, in order to accumulate as much information as possible and to map the key concepts and research gaps. Within this approach, 5 stages, similar to those in systematic reviews, have been described [21].

#### Identifying the research question

Based on preliminary research and the expertise of the research team, the following research question was formulated: ‘What is known in the scientific literature about developing interprofessional shared care plans in chronic care?’ We were particularly interested in the interprofessional issues related to the development of shared care plans.

#### Identifying relevant studies

**Fig. 1:**
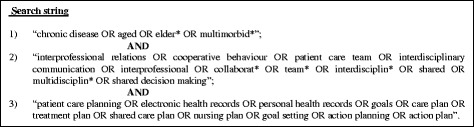
Search string

The search strategy included 3 different concepts: ‘chronic disease’, ‘interprofessional collaboration’ and ‘care plan’ (Fig. 1). Both free-text search terms and MESH headings were used to search the following electronic databases: Pubmed, CINAHL, Cochrane and PsycINFO. The search was updated until April 2014 and limited to human adults and the English, Dutch, French or German language.

In addition to searching electronic databases, the reference lists of relevant articles were checked. Subsequently, we contacted 10 experts in the field, who were asked by e-mail what they regarded as key publications on the topic.

#### Study selection

The selection was made by 2 reviewers (JvD and WE) independently in 3 rounds: first titles were screened, then titles with abstracts, and the remaining set of studies were screened on full text. Any differences and uncertainties were discussed until consensus was reached. Studies fulfilling the following criteria were included: (1) dealing with interprofessional collaboration (2 or more health care professionals from different professional backgrounds) in chronic care for adults; and (2) describing the development of care plans, goals or actions. No methodological criteria were applied, so a broad range of papers, varying from discussion papers to papers based on empirical data, were included. Since the search was sensitive, we also included papers describing interventions in which the development of shared care plans was only a minor element.

#### Charting the data

A descriptive summary of each study was made in a spreadsheet to map the article’s general citation information, methodology and key findings (see additional file 1). Two reviewers (JvD and WE) charted the data independently and discussed the results.

#### Collating, summarising and reporting the results

Initial reading and preliminary content analysis by 2 reviewers (JvD and WE) revealed 3 themes, which were used to structure the findings: the main elements of a care plan, factors influencing the interprofessional development of a shared care plan, and tools to support the building and use of shared care plans. Directed content analysis, using deductive reasoning, was used to validate or conceptually extend the existing preliminary thematic framework described above [22]. Subsequently, the 2 reviewers iteratively extracted the data independently and discussed the results related to these themes until consensus was reached. In cases where no consensus was reached or questions remained, a third researcher (Mv B) was consulted.

## Results

**Fig. 2:**
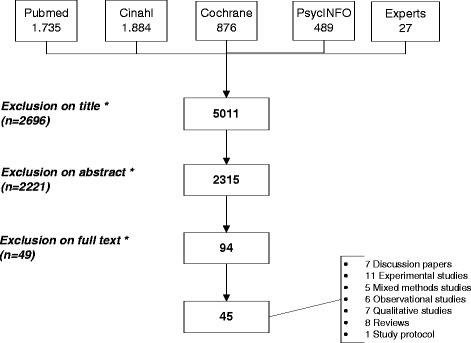
Study selection process

The search resulted in 5011 hits, and after reading the titles, abstracts and full texts and correcting for duplicates, we found 45 articles fulfilling the inclusion criteria (Fig. 2). Of these, 22 had been published in 2008 or later and most originated from the USA (*n* = 15). Eight articles (7 discussion papers and 1 study protocol) reported no new empirical data. In 26 of the other 37, a care plan was the intervention being studied (or part of it), with either quantitative or qualitative evaluations. In addition, 8 reviews were included [14, 23–29]. Most of the reviews aimed to identify models of multidisciplinary collaboration, exploring factors that influence interprofessional teamwork, or assessing the effectiveness of multidisciplinary or collaborative programmes. The review by Dellefield [24] concerned interdisciplinary care planning in nursing facilities, and a written plan of care [24]. Ring and colleagues [28] conducted a systematic review on the use of the asthma action plan [28].

Most of the care plans in the included studies were disease-specific and e.g. related to cancer, pulmonary or diabetes care. Studies used various designs and embraced a wide range of professions and settings, including primary care, rehabilitation, nursing home care, hospital care and home care. Most of the included articles concerned broad topics, including interprofessional collaboration, care planning process, integrated care and teamwork. Only 5 of the empirical studies solely concerned the development and use of shared care plans [17, 24, 28, 30, 31]. Initial reading revealed 3 themes: (1) elements of care plans, (2) factors influencing the development of care plans, and (3) tools to support the building and use of care plans.

### Main elements of a shared care plan

**Fig. 3:**
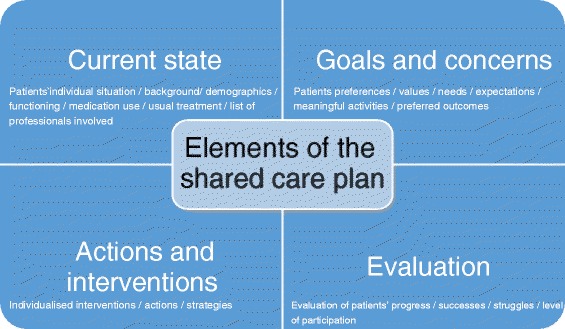
Main elements of a shared care plan

In 18 of the included studies (11 empirical), various elements of a shared care plan were described. However, none of the studies explored ‘elements’ as the main outcome measure. The elements derived from these studies can be divided into 4 categories: information about the current state of the patient; goals and concerns; actions and interventions, and an evaluation of the care delivered and the plan (Fig. 3).

The first element, current state, relates to the patients’ individual situation, and covers information about their background, demographics, functioning, medication use and usual treatment [17, 32]. Besides patient information, the presence of a list of professionals involved was mentioned, with a clarification of their roles and responsibilities [17, 33, 34]. In addition, Chunchu described the current state as ‘about me’, and provided the health care team with essential background information [13]. This current state can be seen as an element of the care plan which is composed and continuously adjusted by the interprofessional team [17].

The second element includes patients’ *goals and concerns* and contains information related to the care requirements and goals formulated by the patient and the professionals. The goals can cover patient’s preferences, values, needs and expectations and can be seen as the central focus of the shared care plan, according to several authors [35–38]. Since setting goals is a complex process and often difficult to understand for patients, Gage [39] preferred to talk about patients’ ‘concerns’ instead of goals [39]. Goals can be explored and described as specific, measurable, assignable, realistic and time-related (SMART) [37, 40, 41]. Furthermore, different levels of goals and behaviour change elements can be distinguished: (1) general goals (e.g. weight loss), (2) ongoing activity (e.g. exercising), (3) specific activity (e.g. walking, swimming), (4) frequency of activity (e.g. 3 times a week), (5) when the activity would occur (e.g. before work), (6) barriers to success, (7) assessment of confidence (1 *low*–10 *high*), and (8) ways to improve confidence [13]. In order to explore personal goals, patients are asked to formulate their own preferred outcomes in the care plan [39]. Patients are also asked to mention activities they enjoy or need to do, also known as meaningful activities [42]. Berger [35] specifically focused on the patients’ own personal stories instead of the illness and used the patients’ own words to describe their goals [35]. According to Berger [35], the care process begins and ends with health care professionals helping patients to explore and tell their own story, including their experience with illness and health [35]. Since the patients’ situation is not static, the shared care plan evolves continuously [17].

The third element concerns the *actions and interventions* that result from the goals and concerns as mentioned in the previous category. Wright [31] stated that a plan of actions is needed, and that the starting point for these actions is the patient’s personal perspective [31]. Dellefield [24] and Metzelthin et al. [42] recommended including individualised interventions (including strategies and actions), tailored to the individual patient, in the care plan, rather than standardised interventions [24, 42]. This will enable the patients’ personal goals to be reached [42]. Redundancy will be minimised when the actions and interventions are specified, time- and date-based and relate to one of the health care professionals involved. Specifying interventions and actions facilitates monitoring and follow-up [23, 42].

The fourth element of the care plan concerns *evaluation,* including the professional documenting the patient’s evaluation of progress, including successes, struggles, the level of participation in goal setting, and capacity to revise the care plan [13, 17, 41]. Gage used the patient’s own outcomes and evaluated these with a format called the ‘tracking and evaluation form’ on 3 dimensions: importance, personal performance and satisfaction [39].

In addition to the elements of a care plan, several authors mentioned preconditions to developing these plans. They stated that the plan should be kept up to date, tailored to the individual patient, and expressed in lay language, balancing the patient’s emotional, social and physical needs. Furthermore, the care plan should be easily accessible to all health care professionals involved [17, 23, 28, 41, 43]. The care plan should also be able to be updated as it travels with the patient, so that the patient does not have to re-explain the situation all over again [17].

#### Factors influencing the interprofessional development of a shared care plan

Factors that influence the process of interprofessional collaboration in developing care plans were mentioned in 35 of the included studies (29 empirical). Often, however, authors did not describe these factors specifically in relation to a shared care plan, but to the underlying process of interprofessional collaboration. We divided these factors into interpersonal factors, organisational factors and patient-related factors.

*Interpersonal factors* are related to individual professionals and interactions between the team members, and concern issues such as knowing each other, the competencies of individual team members [17] and mutual communication [16]. One study identified the need to develop collaboration skills between social and health care professionals in order to better serve the needs of patients with complex health care demands [44]. Professionals in health care teams have various backgrounds and education, resulting in different professional languages and lack of a common vocabulary [45]. These differences have been described as barriers to the negotiation process about a shared care plan [16, 17, 28, 31]. Other factors mentioned in the studies are clarity about and appreciation of each other’s roles and collaboration based on trust and respect [16, 37, 44]. To create a situation of mutual trust and respect, individuals could, according to Lewis et al. [16], focus on reaching team goals instead of individual goals, listen to other team members without attacking each other, convey criticism in a positive way, provide positive feedback and respect, and understand the norms and rules of the team [16].

Most of the factors can be assigned to the category of *organisational factors.* Organisational factors are conditions related to the structure and logistics of team meetings [16, 17, 29] and a shared team vision [37]. Elements related to structure are team composition, division of roles, organisational support and leadership [16, 29, 39]. Preferably, organisational roles and responsibilities should be defined, team members should work towards a common goal, and there should be shared responsibility for optimal patient outcomes [39]. Leadership and coordination were addressed frequently in the studies, and authors often expressed their preference for one person taking the lead in the process [26, 29, 34, 43]. The success of an interprofessional team working according to a structured protocol strongly depends on the person who coordinates the meetings [42]. For both patients and health care professionals, it is often unclear which of the professionals involved has the overall responsibility for coordinating the interprofessional collaboration [44]. Despite the fact that this coordinator could be from any professional background, it is often a nurse (or nurse practitioner) who adopts this coordinating role [13, 14, 24, 29, 34, 42, 46–50]. Elements related to logistics are accommodation, time and place. Time is mentioned several times as a common barrier across different settings and organisations [45]. Pressure of time can be associated with both attendance to meetings and the coordination and development of care plans [16, 26, 32, 36, 44, 45, 51].

*Patient-related factors* regarding the integration of the patient’s perspective during the care plan development process were discussed in 15 studies. These studies described patients’ unique knowledge (experiential knowledge) about their conditions and lives, which complements the knowledge of the professionals. Several authors emphasised that before a team can discuss a patient’s goal, it is essential to know their wishes, expectations and needs [30, 40, 52]. Nine studies highlighted the role of the patient as an active participant in the team, and stressed the importance of patients being empowered by, e.g. providing information, setting goals and developing an action plan [15, 25, 27, 39, 44, 52–55]. However, 4 studies reported difficulties with involving patients in the care process, because of time pressure, unrealistic goal setting, patients’ lack of understanding of the process, leading professional perspective, and difficulties in translating patients’ needs into agreed goals [15, 51, 56, 57].

#### Tools to support the building and use of shared care plans

Fourteen of the selected studies (13 empirical) described tools to support the interprofessional formulation and use of care plans, or tools to support patient involvement. One of the main functions of the tools is to support the exchange of information and bring new information to the team members’ attention through e-mailing or by using an alert system [58–60]. An alert system may be an element of the electronic health record (EHR), sending professionals a reminder to contact colleagues for information and assistance [61]. Likewise, Casas et al. [48] described the use of an ICT platform including a web-based call centre facilitating access by patients, carers and primary care professionals to a specialist nurse who acts as case manager. The use of an EHR, which is associated with greater care coordination among health care professionals and agreement about treatment goals, can be a tool to provide comprehensive patient information [62]. Some of the tools are only used by professionals from one discipline, while others are used by all stakeholders involved [47, 58]. Several professionals use electronic systems before and during the process of developing a care plan [47, 58], while others make use of these systems just to document the care plan [61].

In addition to tools mainly directed at care plan development, several studies discussed ways to empower patients to become involved in the care plan development. The aims of these tools can be divided into developing communication and decision-making skills, training patients and caregivers in mutual communication and decision-making and self-management skills, and training professionals in motivational interviewing [30, 44, 47]. In addition, a user-friendly and patient-centred use of the EHR, in which patients can actively participate, can promote patient self-management [13].

In some cases, patients are able to enter the system and contribute to modifications and adaptations [13, 17]. The option of modifying information can provide the patient with a more active role [13]. Boyd described a tool to enhance this active role of the patient, which merges data from the individual assessments with evidence-based best-practice recommendations to support discussion between professional and patient [47]. Measurement instruments such as the Goal Attainment Scaling (GAS) can be used to evaluate a patient’s progress in terms of attaining personal goals. It is used for patients with multiple complex problems, and monitored during team meetings [39, 56].

## Discussion

This scoping review explored the scientific literature on developing interprofessional shared care plans. This research domain seems to be relatively new as most of the included articles were published in 2008 or later. It is surprising that, despite the fact that the use of shared care plans is recommended in guidelines, the empirical evidence about their value in practice is limited. In most of the included studies, the care plan was part of a larger intervention study. Only 5 empirical studies exclusively concentrated on the development and use of care plans. Most of the care plans in the included studies were disease-specific and focused on, e.g. cancer, pulmonary or diabetes care. Only a small number of studies have addressed the development of shared care plans for patients with multimorbidity [15]. The results of our review identify the different elements of the care plan, the factors that influence the care plan development, including the key role of the patient, and an overview of supporting tools.

Four separate elements of shared care plans could be distinguished from the results: (1) patient’s current state; (2) goals and concerns; (3) actions and interventions and (4) evaluation. Despite the limited empirical evidence, there seems to be consensus among authors about the different elements of a care plan. Patients’ current state includes an overview of the various health care professionals who are involved in the care plan development. However, no studies were found that highlighted the process of decision making as to which professionals should be involved. The exploration of patients’ goals and concerns is essential in the development of a care plan, and can be seen as the central point. The process of patients and professionals collaborating to set goals is regarded as complex and challenging [57]. Lenzen et al. found that this process is influenced especially by attitude, skills and the use of supporting tools [63]. How professionals deal with this complexity in the context of shared care plan development has not been addressed in the included articles, which may either indicate that this is obvious, or can be seen as a blind spot in the research.

We found that the development of the plans can be influenced by factors regarding the interaction between team members, the organisation and facilitation of the care plan development and patient-related factors. This implies that care plan development cannot be seen as a free-standing concept, but more as a result of an underlying process of interprofessional collaboration. In a general reflection on successful collaboration, San Martin-Rodriguez and colleagues studied its determinants and divided them into interactional, organisational, and systemic [19]. Besides the factors we examined, they added systemic determinants, relating to the external environment of an organisation e.g. funding, education and legal and privacy issues. Of the studies included in our review, only the study by Bell et al. mentioned the lack of remuneration for allied care professionals as a barrier to collaboration [46]. Conversely, our study added a category of patient-related factors, highlighting the importance of integrating the patient’s perspective in the care plan. This includes supporting patients’ participation in care plan development, and access to their EHR. Despite the importance of integrating the patient’s perspective in the care plan development process, Dykes et al. stated that to date, care plans have rarely been patient-centred [18].

Some tools are already available to support both professionals and patients in sharing the care plan. Possibilities for and availability of these tools are expanding, partly due to developments in technology, making it possible to share information without personal contact. However, implementation of these tools is often hampered, e.g. by privacy regulations, lack of funding, and the diversity of tools that cannot communicate with each other [18, 64].

The underlying process of interprofessional collaboration seems to be an important aspect in developing shared care plans. Interprofessional collaboration and providing care that focuses on the individual patient’s needs require certain competencies of the professionals involved [65]. Based on a review of different interprofessional competency frameworks, Reeves et al. distinguished core competencies regarding communication, collaboration, patient-centred care, teamwork and the role of a coordinator or leader [66, 67]. Information about crucial skills and competencies could not be extracted from the findings of our review, although we found information about the crucial role of the coordinator.

Some limitations of this scoping review need to be taken into account when interpreting the results. Our search was restricted to a combination of key words based on a preliminary but broad literature exploration. It is possible that this broad topic has caused us to miss key words in our search string, resulting in missing articles. However, by using the input of experts and reference checking, we expect to have minimised this potential shortcoming. Another possible limitation of our study is that we limited our search to databases of peer-reviewed, scientific articles. Books and grey literature were not included. As a result we may have missed relevant publications describing care plans and practical tools. However, among these publications, we do not expect empirical studies with methodologically sound evaluations.

## Conclusions

Research into developing interprofessional shared care plans is rather new. The exploration of the scientific literature identified four topics for further research and implementation in practice. First, more empirical studies of good quality are needed. These studies should focus on the development, use and evaluation of the effects of shared care plans. Second, interventions could be developed to ensure the role of the patient and his/her perspective in developing shared care plans. Interesting interventions to explore include training professionals, enabling patients to access their electronic health records, and translating patients’ goals and concerns into concrete actions and interventions in the care plan. Third, teams considering the use of shared care plans should pay attention to the underlying process of interprofessional collaboration. This includes both interpersonal (e.g. language, interaction, competencies, trust and respect) and organisational aspects (e.g. structure, logistics and the role of a central coordinator / leader). Finally, an increasing number of tools that can be used to facilitate the care plan development process are becoming available for implementation. It is especially linking them to each other which seems to be a challenge.

## Additional file

Additional file 1: Descriptive summary of the included studies per article. (PDF 331 kb)
