# Supplementary material for: Developing interprofessional care plans in chronic care: a scoping review
Source: BMC Fam Pract. 2016 Sep 21;17:137. doi: 10.1186/s12875-016-0535-7 (PMC5031251; doi:10.1186/s12875-016-0535-7)
Supplement: Additional file 1: — Descriptive summary of the included studies per article. (PDF 331 kb) [file 12875_2016_535_MOESM1_ESM.pdf]

Additional file 1 Descriptive summary of the included studies per article

Author	Year	Data-base	Aim of the study	Design	Setting	Country	Key findings
Aberg-Wistedt [52]	1995	Cochrane	The aim of this study was to assess two-year outcomes of patients with schizophrenic disorders who were assigned to an intensive, team-based case management program, and patients who received standard psychiatric services.	Experimental study	Rehabilitation	Sweden	▪ Regular meetings with patients allowed the teams in the intensive case management program to respond flexibly to patients changing needs and supported integration of the activities of the different disciplines on the team<br>▪ First step for patients in the experimental group was creation of rehabilitation plans based on each patient's own wishes.<br>▪ Patients in the experimental condition met regularly with their entire case management team.<br>▪ Regular meetings with patients allowed the teams in the intensive case management program to respond flexibly to patients' changing needs and supported integration of the activities of the different disciplines on the team.<br>▪ A team-based intensive case management model is an effective intervention in the rehabilitation of patients with chronic schizophrenia.
Barnard [57]	2010	Pubmed	The aim of this study was to increase understanding of how a team of professionals and a patient work together to create a set of written goals during the first interdisciplinary, goal-setting meeting.	Qualitative study	Rehabilitation	U.K.	▪ There was rarely a straightforward translation of patient wishes into agreed-on written goals, with the treating team leading goal modification so that goals were achievable. Professionals retained a dominant voice throughout the interaction and the choices they made in their talk directly impacted on the options available to patients for subsequent discourse. Both staff training and patient education are implicated if the aim is to achieve authentic participation of all participants.<br>▪ Goal planning is about much more than performance on a task. The process of goal setting within the overall rehabilitation plan is thought to enhance patient autonomy and interdisciplinary teamwork. Methods are described which ables/unables the patient to be involved in goal setting and strategies used by the treating team to ensure goals were achievable.<br>▪ Goal setting is crucially important within rehabilitation, yet there is very little empirical evidence underlying its practice. Also, various studies of goal setting have investigated participation in relation to predetermined items and generated rich data about how people experience participation, but have not attempted to answer questions about how participation happens.<br>▪ Sharing in decision making improves commitment to the decisions that are made.
Bell [46]	2007	Psychinfo	This study aimed to explore the process of decision making in mental health case conferences involving	Qualitative study	Primary care	Australia	▪ A multidisciplinary meeting comprises two or more health professionals to plan care for a specific person with chronic and complex care needs. Trustworthiness and role specification are significant predictors of collaborative care. Interacting in a structured manner on professional issues

			community pharmacists and primary care physicians.				▪ Lack of remuneration for allied health professionals and the difficulty in arranging mutually convenient times and locations for meetings of three or more health professionals. Also the challenge of communication and joint decision making in health care teams has been cited as a barrier. Further, not involving the patient can be seen as a barrier.<br>▪ An improved understanding of the decision-making process in mental health care conferenced may enable practitioners and policy makers to streamline the process of collaboration.
Benetos [23]	2013	Pubmed	This article offers a pragmatic, simple advice for health care professionals concerning the management of elderly, frail diabetic patients.	Review	Nursing home	France	▪ The foundations stone of a patient's diabetes management is an Individual Care Plan (ICP) expressed in laymans language.<br>▪ The care plan is drawn up after dialogue with the GP, nurses, senior carers and the patient and his or her family.<br>▪ Medical care is organized around specific disease entities, but with frail dependents patient suffering from multiple health problems, the focus switches to a more global approach and clinical decisions are made with the holistic objectives of enhancing quality of life.
Berger [35]	2006	Expert panel	The aim of this study was to evaluate the impact of the new Interdisciplinary Care Plan.	Observational study	Nursing home	Canada	▪ The care plan contains: the goal, the target outcomes (include subjective and objective indicators), the plan (identify responsible team members), the target date(s) and the date met.<br>▪ Clients own story, instead of the illness.<br>▪ Client has to be participant in own care.<br>▪ The care plan has to reflect the clients specific goals and the goals of the team.<br>▪ Develop client goals by using their own words and target outcomes.<br>▪ Goals have to be measurable.<br>▪ There has to be a project leader.
Boorsma [49]	2011	Cochrane	The objective of this study was to determine the effects of multidisciplinary integrated care on quality of care and quality of life for elderly people in residential care facilities.	Experimental study	Nursing home	Netherlands	▪ Multidisciplinary integrated care: 1- : a geriatric multidisciplinary assessment, 2-care plan discussed with the resident, 3-residents with complex needs were scheduled at least twice a year for a multidisciplinary meeting, 4- consultation with a geriatrician or psychologist was optional, 5-date from the Web-based Resident Assessment Instrument was used to provide an overview every three months of 32 risk-adjusted quality-of-care indicators.<br>▪ Nurse-assistants were empowered in relation to monitoring and coordination of care. This aspect has been considered an important ingredient for the improvements.<br>▪ Effect of model: 58,8% of staff and 81,8% of the family physicians felt that their cooperation had improve.
Bovend'Eert [39]	2008	Cinahl	The objective of this study was to describe a practical method of setting personalized but specific goals in rehabilitation that also facilitates the use of goal attainment scaling.	Discussion paper	Rehabilitation	U.K.	▪ It is essential to know what the patient's wishes and expectations and goals are and to know enough about the patient's situation to allow the team to set valued and achievable goals.<br>▪ In this article, a new, structured method for writing goals that are specific and measurable without too much effort is described. it is also flexible enough to cover most situations, it is patient specific and it can easily been taught and used by the whole team.

							▪ Goals should be: relevant to the person concerned, should be challenging but realistic and achievable, and should be specific (in order to measure them). All people should agree on the goals, on the methods to be used to achieve these goals and on each person's role in the process.<br>▪ Benefits: It is effective, and creating interdisciplinary goals improves the collaboration of the various disciplines and creates clear aims for the patient and the disciplines.
Boyd [47]	2007	Psychinfo	The purpose of this study was to test the feasibility of a new model of care designated to improve the quality of life and the efficiency of resource use for older adults with multimorbidity.	Experimental study	Primary care	U.S.A.	▪ Guided Care: a registered nurse completes an educational program and then uses a customized electronic health record in working with 2 to 5 primary care physicians to meet the complex needs of older patients.<br>▪ Chronic Care model: Improve clinical and /or financial outcomes, delay nursing home placement, improve caregivers well-being, reduce burden, improve quality of life, reduce use of hospital. Improved the quality of care, especially the communication & coordination among providers.<br>▪ The guided Care model does not require deep organizational or structural change in the existing health care delivery system, nor does it require ill older people to change primary care facilities.<br>▪ Qualities that make registered nurses well suited to becoming GCNs include proficiency in communication, flexibility in complex problem solving, cultural competence, comfort with interdisciplinary team care, experience in geriatric and community nursing and enthusiasm for coaching patients and caregivers in self-management.
Casas [48]	2006	Psychinfo	The aim of this study was to examine whether a simple and well-standardized, low-intensity integrated care intervention can be effective to prevent hospitalizations.	Experimental study	Integrated care	Spain & Belgium	▪ ICT platform including a web based call center.<br>▪ Measures: re-hospitalization was assessed through hospital records and systematic questionnaires on use of healthcare resources administered to patients. Phone and personal interviews.<br>▪ Integrated care showed a lower hospitalization rate and a higher percentage of patients without re-admissions than usual care without differences in mortality.<br>▪ It effectively prevents hospitalizations for exacerbations in chronic obstructive pulmonary disease patients. It resulted in enhanced self-management of the disease together with a higher accessibility to healthcare professionals. An early detection and better management of exacerbations . Better health related quality of life.<br>▪ Barcelona: key role for the specialized nurse.<br>▪ Leuven: Key role for the GP.
Chunchu [13]	2012	Psychinfo	The aim of this study was to evaluate whether a team approach to using an EHR based patient centered care plan (PCCP) improved collaborative self-management planning.	Experimental study	Primary care	U.S.A.	▪ PCCP model comprises three sections: 1)'About me': provides the health care team with information about the patient's preferences, values, and includes an area for the patient to list other providers the patient views as part of his care team, 2) 'My goal': prompts a collaborative discussion between team members of a health care team and the patient using a problem solving approach to refine goals into a manageable action plan, and 3) 'My Progress': which is

							used during the follow-up visits or phone calls to track successes, to examine struggles and to stimulate collaborative decision making between team members and the patient.<br>▪ The PCCP model can help team members to engage patients with chronic illnesses in goal setting and action planning to support self-management<br>▪ Health care systems with integrated medical records could use designs like the PCCP to help patients feel known across primary care, specialty and inpatient settings.<br>▪ Patients want to be known as a person with individual needs and values.<br>▪ Patients and physicians recognize the medical assistant/nurse-physicians as part of team in fulfilling the purpose of the PCCP. Training them to use a patient centered care plan in the HER is a relatively simple intervention changing the content of patient interactions through improving patient engagement.<br>▪ Patients and providers may not have a shared language or conceptual understanding about health care goals setting; therefore, training needs to address patient understanding and receptivity for creating health goals.
Clausen [17]	2012	Pubmed	This article described the development of an interprofessional care plan for an older adult woman with breast cancer.	Qualitative study	Acute care	Canada	▪ Little is known about developing interprofessional care plans, particularly in oncology. Need exists to understand how multiple voices can be integrated into a shared vision for collaborative patient-centered care. Most literature describes a multidisciplinary approach where each clinician uses a care plan individually to address and document patient care issues form their uniprofessional perspective. Literature describing the process of developing an interprofessional care plan is scant. No literature was found demonstrating the involvement of a team of cancer care profs with various professions and specialties in the initial development of the care plan itself.<br>▪ Shared care plans are a means to improve communication across disciplines, strengthen and document coordination of services and improve continuity of care for patients. It facilitates and documents a process of shared decision making among a team of healthcare professionals, where roles and responsibilities are defined, team members are working toward a common goal and responsibility for optimal patient outcomes is shared. that interprofessional approach is based on the integration of knowledge and expertise of each profession and understanding and translating those various professional perspectives to achieve the common goal. The interprofessional care plan should highlight the process of care, rather than being solely a linear chronology of interventions or tasks. It should identify important check-points for evaluating patient comprehension. Capturing multiple professional and patient voices in all stages of development.

							▪ It was important to formalize interprofessional discussion in the interprofessional care plan. Highlighting the diagnostic, treatment, and follow-up phases of care. The care plan must be open and generic, allowing it to be tailored to each specific setting as well as having evidence-based key variables that impact the patients decision making and ultimate health outcomes. A culture of collaboration is necessary to make it effective. the information in the profile should be accessible to all healthcare profs involved in the patients case. The picture of the patient is not static, but continually evolves as information is gathered by different professionals throughout the care experience.<br>▪ Shared care planning is a pivotal part of interprofessional teamwork; however, individual team member competencies shape the efficacy of the process. Therefore developing an effective care plan is part of a process that also creates and nurtures a culture of collaboration among healthcare providers.<br>▪ Use of an electronic case to facilitate collaboration and dialogue. Furthermore, the model itself was suggested as a possible framework for a cancer center's electronic health record. Allowing each professional involved to have the most current information and add or modify information as necessary, with some modifications possibly being done by patients themselves. Such an EHR could be valuable in reducing overlaps and gaps in care.
Clay [37]	2003	Cinahl	The aim of this article is to examine key factors relating to the rehabilitation of older people.	Discussion paper	Rehabilitation	U.K.	▪ Collaboration is key to success of interdisciplinary working. The whole team agrees goals with the client and therefore avoids duplication or conflict in goals.<br>▪ The blurring of professional roles while preserving the separate identity and expertise of individual professions, will lead to greater synergy, so that more comprehensive outcomes are achieved.<br>▪ Collaboration requires members to work together with shared philosophies as well as goals, having mutual respect and trust and a clear understanding of the expertise of each discipline and team member.<br>▪ The key attributes of the nurses working in the field of rehabilitation: to work cooperatively with the interdisciplinary team, being optimistic and creative as well as having a broad knowledge base.<br>▪ Goals should have particular 'smart' characteristics.<br>▪ Practitioners who are new to goal planning need to feel supported and should be given the opportunity to reflect, discuss, debate, question and learn.<br>▪ The goals and desired outcomes for each individual will be unique. Goal planning should be part of the overall care plan in which the clients own values, beliefs and aspirations are recognized and valued, and form the central focus of the rehabilitation process.<br>▪ Advantages of patient- centered, interdisciplinary goal planning include: encourages members of the rehabilitation team to work

							together, facilitates partnership working with the older person and their family, provides a structure for the rehabilitation process.<br>▪ If there is effective co-ordination and communication, then the patient and family will be active participants in the rehabilitative process.<br>▪ Nurses have an unique place within the rehabilitation team. Their 24-hour contact with patients, seven days a week, provides a chance to use this time therapeutically. The nurse is the communication link between all those involved. Nurses need a wide ranch of skills and competencies, including: continuing education, professional and practice development, reflective practice and experience.
Counsell [61]	2006	Cinahl	The purpose of this article was to describe the Geriatric Resources for Assessment and Care (GRACE) clinical intervention that was tested in a randomized clinical trial.	Experimental study	Primary care	U.S.A.	▪ GRACE-intervention: upon enrollment, the GRACE support team meets with the patient in the home to conduct an initial comprehensive geriatric assessment. The support team then meets with the larger GRACE interdisciplinary team to develop an individualized care plan. The support team then meets with the patients primary care physician (PCP) to discuss and modify the plan. The support team then implements the plan.<br>▪ The team completes special training in implementing the GRACE protocols and working as an interdisciplinary team during 12 weekly small group seminars.<br>▪ GRACE team meets face to face with de patients PCP to review a computer-generated summary of the patient assessment and team suggestions.<br>▪ Physicians were satisfied with the intervention and the resources available, somewhat to very helpful in providing care to older patients, 'just right' implying that the supplemental care provided by the GRACE support team was neither insufficient to meet patient needs nor excessive or duplicative.<br>▪ Patients and their PCPs were highly receptive to this new model of care.<br>▪ It is hypothesized that the intervention will result in better health status, greater functional independence, fewer emergency department visits and hospitalizations, fewer nursing home days over 2 years of follow-up. Potentially cost-effective.<br>▪ Support of an electronic medical record and longitudinal tracking system: the GRACE support team provides ongoing care management and coordination of care across multiple geriatric syndromes, providers and sites of care. Hospital receive automated prompts via the electronic medical record system to contact the GRACE support team for information and assistance with follow-up and coordination of care.
Crossen-Sills [30]	2007	Cinahl	The reported case study in this article highlighted how veritable coordination of services, designed to meet the immediate and long-term	Observational study	Home care	U.S.A.	▪ A preliminary family meeting was conducted to develop a joint plan. This plan would focus on the steps that both the family and home care agency would need to take to provide a safe discharge to home. The family had the option to accept, modify or reject the plan. All

			needs of a family, were achieved through creative problem solving.				▪ clinical specialists required for planning and coordination of care were present and involved in planning process.<br>▪ Weekly case conferences conducted with all members ensured consistency, follow-through and success with the plan. Communication and coordination- all professionals to be aware of the complex aspects of care. To address issues before they became problems. Coordination around the same goal.<br>▪ Grand rounds, as an opportunity to share best practices, and to meet and share a number of home care issues.<br>▪ A plan was established that spaced the clinicians visits throughout the week, ensuring that the patient would not be overwhelmed with too many professionals coming on the same day. The clinical interventions were designed on the basis of the patient's goals and medical needs.<br>▪ Throughout the planning process, every effort was made to encourage the patient to express his desires and needs.
Dellefield [24]	2006	Expert panel	This article is a critical review of the history, research evidence and state-of-the-art technology in interdisciplinary care planning and the written plan of care in American nursing homes.	Review	Nursing home	U.S.A.	▪ The Resident Assessment Instrument/Minimum Data Set (RAI/MDS) provide the core framework for the development of interdisciplinary care planning and the written care plan.<br>▪ A care plan must include all documented interdisciplinary assessments and progress notes, which will reduce duplicative charting. The redefined care plan would include a listing of the headings of the triggered RAPs for which care plans will be written, as well as other clinical priorities identified during the interdisciplinary assessment process. The specific content of the care plan that accompanies each listed item would be developed with the assumption that all clinician have access to the clinical record, composed of interdisciplinary assessments and progress notes and that includes the specific objectives, time lines, and clinical services offered.<br>▪ The care plan provides individualized interventions for residents (often standardized). But standardized interventions are best described in policies and procedures and job descriptions, rather than in written care plans.<br>▪ Only information about technical or interpersonal care processes unique to the resident that all clinicians need to know to efficiently and effectively provide services on a 24-hr basis has to be included in the written care plan.
Duff [25]	2009	Cinahl	This article aims to emphasize the fundamental aspects of the process of goal planning and references the key literature in the arena.	Review	Rehabilitation	U.K.	▪ Assessment and rehabilitation requires the knowledge and skills of an interdisciplinary team in partnership with the client (proactive involvement). A comprehensive and holistic approach to assessment that is team focused, specific and inclusive of the patient concerned. Each member brings his or her professional knowledge to the task.<br>▪ Team approach requires each member of the team to have an accurate understanding of each other's roles and responsibilities.

							▪ Goal setting theory (goals affect action): It points to the need to include the patients at all levels of planning in order to achieve reliable behavior change and to maintain the change.<br>▪ A team approach needs to be highly structured and coordinated, this allows members of a team to identify common goals. *The needs assessment checklist (NAC): Developed through consultation with patients and the interdisciplinary team. The setting of goals is directly derived from the assessment tool, and goals are set between the patients and the interdisciplinary team in a collaborative relationship. *A structured and focused meeting<br>▪ No one professional group can meet all of the needs of these patients. Each professional brings his or her specific area of expertise to the assessment process but needs to work in conjunction with the others to practically solve the difficulty. Goal planning can be effective in resolving conflict among team members because the focus is on client-driven goals.<br>▪ Involvement client: feeling a sense of control over their new life circumstance and, in time, to developing the self-management skills essential for resuming a satisfying life. Patients improve adherence and healthful behaviors, improved health status and decreased days in hospital. *The team approach sends a strong and positive message of consistent management to the patient.<br>▪ Central role of the patient in the rehabilitation process. Participation stimulates information exchange.
Duner [33]	2013	Cinahl	The aim of this study was to examine professional collaboration and professional boundaries in interprofessional care planning teams.	Observational study	Integrated care	Sweden	▪ Decision-making in both teams was more or less profession specific. In the investigation & assessment phase, integration between professionals was most noticeable. The home care planning team tended to work in a more integrated manner.<br>▪ Work with a greater degree of integration and collaboration then in multiprofessional teams. The professionals interact and adjust to each other. To achieve a comprehensive assessment from different professional perspectives leading to proper health and social care corresponding to the needs of the care recipients.<br>▪ Striking a balance between their specific professional roles in the team and the role of the team as a whole. Working together can lead to more security in one's professional role, when one sees the competence of the other team member and can concentrate on one's own expert knowledge.<br>▪ Clarifying the roles of all professions concerned with needs assessment and care planning for older people, so that each can contribute effectively in the best interest of the service recipients.<br>▪ Professionals learned from each other, deepened their knowledge and improved assessment.<br>▪ Home planning team: important role for nurse who was appointed as case manager. Discharge planning team: Social worker.<br>▪ There's a general lack of studies based on observations of interprofessional teamwork combined with interview data.

Eleazer [55]	1994	Pubmed	The aim of this article was to provide historical background on the PACE project, describe the model of care implemented in PACE programs, and provide an update on Palmetto SeniorCare.	Experimental study	Integrated care	Colombia	▪ The PACE (Program for All-inclusive Care of the Elderly); Individual assessment by team members, After this: The entire interdisciplinary team gathers to discuss and develop a care-plan for the participant.<br>▪ Can lead to reductions in morbidity for the participant. Increased social time, improved their family rime and resulted in reduced amounts of stress. 90%: Quality of life improved.<br>▪ The PACE/On Lok model with its focus on multidisciplinary assessment, coordination of services and participant needs.<br>▪ The coordination which characterizes PACE care is developed through constant attention to effective group process wihtin the team. -Frequent team meetings, extensive contacts among team members, participants and caregivers.<br>▪ Participants and caregivers are involved in all phases of this process
Eloranta [44]	2008	PsychInfo	Aim of this study was to describe the experiences of multiprofessional collaboration in promoting personal resources among older home care clients (75 + years).	Qualitative study	Home care	Finland	▪ This study revealed the need for developing collaboration skills between social and health care professionals so that the staff serve the needs of aged clients better together.<br>▪ Effective multiprofessional collaboration demands of the common goals for care. Multiprofessional collaboration increased with determining to whom the main responsibility for the coordination of collaborative coordination belonged.<br>▪ Common goals, good communication, mutual respect for all the professionals and equal value placed on their contribution to existing collaborative practices.<br>▪ For client participation, the following skills are required: communicaton and decision -making skills.<br>▪ Clients were uncertain which professional had the overall responsibility of coordinating the multiprofessional collaboration. 'It would be nice if there was one contact person who I could talk to".<br>▪ Lack of time, different orientations towards client care between health and social care professionals were perceived as barriers.
Emery [58]	2012	Pubmed	The purpose of this study was to demonstrate the feasibility of the BRIGHTEN Program (Bridging Resources of an Interdisciplinary Geriatric Health Team via Electronic Networking), an interdisciplinary team intervention for assessing and treating older adults for depression in outpatient primary and specialty medical clinics.	Experimental study	Integrated care	U.S.A.	▪ The BRIGHTEN intervention contains the following components: screening, assessment, virtual team communication, participant-centered treatment plan development and connection to recommended evidence-based services.<br>▪ Measures: to build a cohesive, interdisciplinary virtual team with members who provided strong evidence-based treatment recommendations and were able to have open constructive discussions about older adult participants.<br>▪ Goals were met and team members learned from each other's recommendations, which enhanced their work with BRIGHTEN and non-BRIGHTEN patients. Email communication also maximized efficiency of team participation (time). Other outcomes: improvement in depression symptoms and general mental health. Also, patients find it more palatable to go to a program instead of a Psychiatrist. Bread team based intervention is more inviting than an

							exclusive mental health team. Also, feeling comfortable to have a full team of providers to count on.<br>▪ Virtual collaboration; virtual communication without the in-person meetings. E-mail communication.<br>▪ Virtual Integraded Practice (VIP) developed communication protocols via email, phone and fax to maximize collaborative care for primary care providers with community providers.
Gage [38]	1994	Expert panel	Given the paradigm shift to patient-focused care, the Patient-Driven Interdisciplinary Care Plan is the next, natural evolutionary step for the health industry. The plan, based on four underlying principles along with preliminary impressions of staff members and patients on one pilot unit are presented in this article.	Discussion paper	Chronic care	Canada	▪ The reality of the patient is the central focus of the interdisciplinary care plan. First, patients concerns are documented in the Concerns Box. After this, the primary care worker develops (together with the patient) statements (measurable and time-limited) of the desired outcomes. After this, the statements are transferred to the Tracking and Evaluation Form, that includes a Type Column (who generated the desired outcome), an Initials Box (person documenting the outcome), a patient rating of each statement following three dimensions (importance, performance and satisfaction). There is also space where the primary care worker can document the patients level of participation in goal setting. In the end there is some space to document patients strength with respect to the attainment of the desired outcomes.<br>▪ The statements of objectives (measurable and time-limited) are documented on the Discipline Tracking Sheet. The relationship of each objective (useful interventions, discussed between the patient and the professional) is shown by placing the reference number of the desired outcome (obtained from the tracking and evaluation form) into the '#' column on the discipline tracking sheet. The third column includes the date of achievement.<br>▪ Each professional discipline addresses one aspect of the needs of the patient, just as each factory worker performs one aspect of the assembly process.<br>▪ The time has come to involve the patient as a fully functioning member of the interdisciplinary care team.<br>▪ In the current multidisciplinary model, the professional disciplines function almost independent of one another, which can lead to the patient or family receiving conflicting messages from different team members.<br>▪ The presence of a hierarchy, which gives more power to some disciplines than to others, also serves to increase the possibility of conflict.<br>▪ Teams that reach a consensus on the goals of treatment find that team members are more supportive of each other.<br>▪ Goal-attainment scaling is one popular way to measure change at a patient-specific level.<br>▪ The word 'goal' is not mentioned to the patient. Goal setting is a sophisticated process that is difficult for many patients to understand. However, patients do know what their concerns are and do have ideas for how these concerns could be addressed.

Graetz [62]	2014	Pubmed	The aim of this study was to examine whether primary care team cohesion changes the association between using an integrated outpatient-inpatient electronic health record (EHR) and clinician-rated care coordination across delivery sites.	Experimental study	Primary care	U.S.A.	▪ EHR can be a tool to provide comprehensive patient information.<br>▪ The shared use of an HER across hospitals, specialist and primary care practices, and other provider organizations offers great potential to improve coordination by enabling access to comprehensive, current patient information each time a patient is seen by clinicians.<br>▪ The association between EHR use and reported care coordination varied by level of team cohesion. EHRs may not improve care coordination in less cohesive teams.
Hedrick [60]	2003	Cochrane	This study aimed to compare collaborative care for treatment of depression in primary care with consult-liaison (CL) care.	Experimental study	Primary care	U.S.A.	▪ A mental health team provided a treatment plan tot the primary care provider, telephoned patients to support adherence to the plan, reviewed treatment results and suggested modifications to the provider. Differences with the consult-liaison group (control): the systematic formulation and communication of a treatment plan and the systematic evaluation of patient progress and subsequent plan reformulation.<br>▪ Collaborative care: includes patient-, provider- and system-level components based on a chronic illness model.<br>▪ Collaboration between primary care providers and mental health specialists can improve depression treatment and supports the necessary changes in clinic structure and incentives. Thus: relatively small changes in the organization and delivery of mental health services can provide measurable benefits, even for a very difficult patient population.<br>▪ Treatment options were selected in a stepwise fashion beginning with the least resource-intensive option consonant with previous treatments and the patients preference. if the 6- and 12- week progress evaluations indicated the option was ineffective, a new or stepped-up option was recommended.<br>▪ The team communicated with primary care providers using electronic progress notes. The system incorporated an alert and co-signature function that brought any new communication to the providers attention and enabled the team to track receipt and acknowledgement of notes and follow-up.
Johansson [43]	2012	Pubmed	In this article, the authors focused on the vital importance of a comprehensive care plan for care coordination.	Discussion paper	Rehabilitation	U.S.A.	▪ Key to managing complex cases is a professional case manager who works closely with a multidisciplinary team. When complex cases occur, a multidisciplinary team will be put together, the case managers has the central role of coordination and has to foster the collaboration.<br>▪ The care plan must address the holistic needs of the patient, including physical and behavioral health, ability to provide self-care, support for the family, and safety of the home environment, as well as access to resources in the community.<br>▪ At the heart of the care plan are the goals set by patients and their families.<br>▪ At the center of the team is the professional case manager who facilitates communication and fosters collaboration.

							▪ Rehabilitation underscores the importance of a multidisciplinary approach. When complex cases are involved, a care team is put together that includes physicians, nurses, other clinicians, rehabilitation professionals, including occupational therapists, physical therapists and speech therapists.
Johnson [50]	2012	Cochrane	The objective of this study was to comprehensively evaluate a collaborative care model for comorbid depression and type 2 diabetes within a Canadian primary care setting.	Study protocol	Primary care	Canada	▪ TeamCare, the Collaborative care intervention, comprising a nurse care manager guiding patient-centered care with family physicians and consultant physician specialists to monitor progress and develop tailored care plans. The nurse care manager will remain responsible for final treatment discussion.<br>▪ The control group contains usual care, without additional active support from the CM.<br>▪ The CM will have weekly meetings with the consulting specialist to review new cases and patient progress, and then communicate team treatment recommendations to the primary care physician. A treat-to-target/stepped care approach will be used at each phase of the intervention. Recommended treatments have been based on algorithms, developed in collaboration with the PCN's by compiling various guidelines and sources.
Keks [34]	1998	Pubmed	This article is a discussion paper on collaboration between general practice and community psychiatric services for people with chronic mental illness.	Discussion paper	Primary care	Australia	▪ At a case conference meeting, the patient's problems and needs can be considered within that individuals context and preferences, and matched with available treatments, professionals, services and resources.<br>▪ Elements of a shared care plan are specification of the personnel responsible, the responsibility of the various parties, the plan for handling crisis, and date of review.<br>▪ The best kind of collaboration occurs when general practitioners and community psychiatric service staff get to know about each other's roles and stay in personal contact.<br>▪ Because of the complexity involved, it may be necessary for collaborative relationships to be supported by a coordinator.<br>▪ Strategies that facilitate communication and common understanding between GP and staff in community psychiatric services are essential for collaborative care.<br>▪ Case managers coordinate the care for patients who require a number of services from different providers.
Legaré [45]	2013	Cinahl	The aim of this study was to evaluate health professionals' intentions to engage in IP-SDM in home care and explore the factors associated with this intention.	Mixed method	Home care	Canada	▪ An interprofessional team collaborates in identifying best options and helps patients determine their preferences, enabling them to take more control over the treatment plan. Collaboration among various health professionals.<br>▪ The mean intention to engage in IP-SDM was positive. The factors influencing the intention vary across types of providers: The implementation intervention would need to be tailored to each group of providers even if they work together as a team. Also, this study confirmed that perceived behavioral control is the factor most closely associated with intention to engage in IP-SDM. Participants proposed appointing facilitators who could help implement IP-SDM

							in the home care teams. They suggested involving all professionals from the outset in the management of a case and providing tools for singling out cases for which an IP-SDM approach is appropriate.<br>▪ Planned team meetings, better team cohesion and shared work methods could facilitate the implementation of IP-SDM.<br>▪ Lack of time, difficulty of coordinating professionals, failure to synchronize their interventions in the patients care, lack of human resources and high staff turnover. Lack of cohesion among members and different work methods, no common vocabulary.<br>▪ The IP-SDM model has the potential to help overcome many of the identified barriers with only slight adaptations to each organizational structure.
Lewis [16]	1998	Pubmed	This paper describes a pilot team development intervention that was designed and implemented by an operating psychiatric team in a geropsychiatric hospital, to improve the interdisciplinary treatment planning process.	Observational study	Acute care	U.S.A.	▪ A broader view of collaboration is that it includes merging the expertise and perspectives of professionals with different backgrounds.<br>▪ An interdisciplinary team brings together diverse skills and expertise on the assumption that this will result in more effective, better coordinated, better quality services for clients.<br>▪ In all cases of initial disagreement, the team reached a consensus through discussion. All team members voiced their opinion that it was helpful and worthwhile. These changes resulted in more complete treatment plans and more adaptable to patient needs.<br>▪ The meetings should be such that members are comfortable concerning their participation and the team should recognize the contributions of individuals<br>▪ Respect, share responsibility, be willing to trust and compromise, assume responsibility for their own actions, focus on reaching team goals, listen to others without attacking, convey criticism in a positive way, provide positive feedback and understand the norms of the team such as being on time, have been described as important competences related to interprofessional collaboration.<br>▪ Using individuals who are not members of the team as observers can help the team by providing objective feedback, and by serving as catalysts to promote open discussion. Outsiders are free to concentrate on observing and thus often notice behaviors that may not be apparent to members. They can help the team identify processes that can be improved.<br>▪ For a team to improve, it is important to prioritize a few target changes at any one time.<br>▪ Reducing the average time required for the meetings, a carryover effect, improvement of attendance of members, more efficiency in preparation.<br>▪ Possible barriers to team functioning are: different training, perspectives, values, languages, working styles, turf battles and competition, failure in ongoing communication, and a limited amount of time.

Martin [26]	2010	Pubmed	This article provides an overview of the evidence base for interprofessional collaboration involving doctors and nurses and new models of care in relation to patient outcomes.	Review	Integrated care	Switzerland	▪ Interprofessional collaboration occurs when 2 or more members of different healthcare professions work together jointly to solve problems or provide services. It is essential to facilitate information flow and the coordination and provision of healthcare within an increasing diversity of disciplines where one health professional can no longer meet all patient needs.<br>▪ Collaboration requires shared power and authority, based on knowledge and expertise and an interaction between subjects with trust, mutual respect and joint contributions to a common goal.<br>▪ Trust, respect, shared leadership, recognition of unique contribution, collegiality and open communication.<br>▪ Enablers for collaboration: joint nurse/physician practice committees, integrated patient records, joint practice record review, and the use of protocols or critical pathways in the care of specific patient groups.<br>▪ Barriers to interprofessional collaboration: time pressure, lack of explicit descriptions or of understanding of each other's roles and tasks, poor organizational support, absence of clear leadership, different traditions and professional values, different aims and priorities, and vertical management structures with discriminatory power structures.
Metzelthin [41]	2013	Pubmed	The aim of this study was to examine the extent to which the interdisciplinary care approach is implemented as planned and to gain insight into healthcare professionals' and frail older people's experiences regarding the benefits, burden, stimulating factors and barriers.	Mixed method	Primary care	Netherlands	▪ Frail older people felt acknowledged by healthcare professionals and experienced support in handling their problems and fulfilling their wishes.<br>▪ Frail older people often have complex healthcare needs, which have to be addressed by various healthcare professionals.<br>▪ The roles in the team were clearly defined according to the healthcare professional interviewed. Overlapping tasks were minimized by formulating an integrated treatment plan, involving collaborative goals and specified tasks for each discipline.<br>▪ Frail people had to think about their individual needs, preferences, concerns and problems. This information was used to develop a preliminary treatment plan, including individual goals, strategies, and responsibilities.<br>▪ Sharing information during the team meetings led towards a better understanding of concerns, problems and wishes of frail older people. Information acquired was relevant for the delivery of tailor-made care.<br>▪ Healthcare professionals also learned much about each other's expertise, which led to more consultations of involved healthcare professionals and more frequent referrals.<br>▪ With regard to integrated care, nurses are recommended as case managers to plan, organize, and monitor the care process and to facilitate cooperation between professionals.
Mitton [59]	2007	Cinahl	The aim of this project was implement and evaluate a collaborative partnership	Mixed method	Primary care	Canada	▪ The partnership improved accessibility and availability. Anxiety (patients/caregivers) decreased and reduction of hospital admissions. Decreased costs and high level of satisfaction.

			between homecare nurses and family physicians in the rural Alberta.				▪ Successful partnership: professionals willingness to participate, their predisposition to collaboration and regular face-to-face communication.<br>▪ The electronic patient health record system was modified to enable nurses to access patients records and communicate with the physician remotely form the point of care (EMR).
Newbould [15]	2012	Expert panel	The study explored experience of care planning in patients with long term conditions in three areas in England.	Qualitative study	Primary care	U.K.	▪ In this predominantly frail elderly population, care planning was not a discrete event with an explicit discussion leading to a written care plan, as envisaged by policy.<br>▪ The process of care planning may result in the output of a care plan, a written document summarizing discussions and setting out agreed actions and goals.<br>▪ To date much care planning work in the UK has been disease specific, and there is little evidence on the implementation or function of care plans for patients with more complex needs.<br>▪ Patients should participate in one or more explicit care planning discussions, covering the setting of goals, provision of information, support for self-management, agreements on treatment and medications, action planning, support services available and plans for future reviews.<br>▪ Only three participants mentioned that goal setting and action planning were discussed at all in relation to their care.
Norburn [36]	1995	Cinahl	The purpose of this project was to develop and implement a collaborative planning process to enhance humane end-of-life care for nursing home residents.	Qualitative Study	Nursing home	U.S.A.	▪ Model of individualized planning and interdisciplinary collaboration. Move from an acute care model to a caring psychosocial model<br>▪ Cooperation, communication, respect, good attitudes, carrying your share of the load, working hard, setting goals, and leadership are described as elements related good teams.<br>▪ More positive approach, one characterized by ongoing dialogue with the resident and family to determine their values and the care they wanted.<br>▪ The existing care planning focused on meeting federal regulations, not on individualized resident care. The existing plans appeared to be computer-generated and often failed to reflect individual resident needs.<br>▪ The difficulty or even impossibility of substantially changing 'nursing care plans' without doctors' orders is a practical hindrance to timely changes.
Oeseburg [32]	2004	Cinahl	The goal of this study was to decrease the discrepancies between needs and the use of health care services by means of the transmural care model for MS patients (TCMMS) and to test if the TCMMS is applicable in practice.	Mixed method	Home care	Netherlands	▪ Mutual agreement, cooperation and shared responsibility are recognized as ingredients of interprofessional competences.<br>▪ Using the patient care file was time consuming (because the file was used in addition to other existing dossiers). Also, transfer of information between the professionals was scarce. It was not easy to contact some health care professionals and some were reluctant to cooperate with the NS because coordination was traditionally a task of the GP. Also, health professionals in the Netherlands aren't

							trained, in their initial education program or on the job to cooperate with each other.
Orchard [54]	2012	Cinahl	The aim of this study was to test and evaluate the Assessment of Interprofessional Team Collaboration Scale (AITCS)	Experimental study	Primary care	Canada	▪ Interprofessional collaborative practice (IPCP) is defined as 'a partnership between a team of health professionals and a client in a participatory, collaborative and coordinated approach to shared decision making around health and social issues'.<br>▪ Coordination (the ability to work together to achieve mutual goals), cooperation (ability to listen to and value the other viewpoints and contribute your own), shared decision making (working together) and partnership (open and respectful relationships, work equitably together to achieve shared outcomes).<br>▪ Patients need to be involved in their own health care. Many health professional teams are reluctant to include patients and family members as full members of the team. An interprofessional team cannot be considered truly collaborative unless it includes patients and family members as full members of the team.<br>▪ Role clarification, role valuing, creating trusting relationships that lead to shared decision making, and power sharing can be considered facilitators. Practitioners need a better understanding of what; interprofessional collaboration' means and how it is practiced; organizations need to support change toward collaborative practice including provision of continuing education.<br>▪ Most research focus on team effectiveness without attention to the evolutionary process underlying health care team practice in institutional settings or individual performance assessment at organizational levels. There is also an absence of research focusing on the role of patients within collaborative teams. * Many health providers believe they currently function as interprofessional collaborative teams, when in fact their practice remains multidisciplinair.
Ospina [27]	2003	Cochrane	The aim of this study was to evaluate the current published scientific evidence on efficacy, effectiveness, and economic consequences of multidisciplinary pain programs (MPPs) for patients with chronic pain.	Review	Chronic care	Canada	▪ In terms of functional improvement and pain reduction. All the reviewed CPGs recommend the use of an interdisciplinary/multidisciplinary team approach that includes physicians, psychologists and physical/occupational therapists.<br>▪ Multidisciplinary pain programs are defined as being a comprehensive approach that involves coordinated intervention among a variety of disciplines working together in the same facility in an integrated way with joint goals and with ongoing communication.<br>▪ A potential shortcoming is that access to a range of health-care providers is usually limited and that the patients care is rarely coordinated, even when the program considers itself to be interdisciplinary-grounded.
Preston [53]	1990	Pubmed	The aim of this article is to describe and understand	Observational study	Home care	U.S.A.	▪ A client who requires rehabilitation is likely to have complex physical, emotional, and psychosocial involvement. Since

			rehabilitation using a team approach				professionals of no one discipline have the expertise to manage all of these areas, a variety of disciplines is needed.<br>▪ The client, family and significant others are considered to be part of the team. Although each professional team member will focus primarily on his or her own discipline's area of expertise, all members need to consider the larger picture.<br>▪ Effective team functioning requires continuous, open communication, preferably carried out as a group process and with written documentation. Since each discipline focuses on just one aspect of the client, the only way to seen the entire client is to combine data from all members. The goals of all team members must be consistent with one another and have the full support of everyone.<br>▪ The duties of the rehabilitation team's case manager should be clearly specified and the individual chosen to be case manager should be recognized as such by the other team members.
Ring [28]	2011	Expert panel	The objective of this study was to better understand what helps and/or hinders asthma action plan use from the professionals and patients/carers perspective.	Review	Chronic care	U.K.	▪ An action plan is defined here as 'a written or electronic record agreed between an individual patient and their health professionals, providing personalized guidance on what actions to take when their asthma deteriorates.<br>▪ Action plans require to be tailored to the needs of patients/carers, better targeted to potential users, and addressing the broader issues of living with a long-term condition.<br>▪ Differences in the language used by patients carers and professionals to describe asthma and the different meaning ascribed to asthma management and asthma control are perceived barriers.<br>▪
Russell [51]	2008	Expert panel	This study investigated the experience of family physicians and patients with a chronic illness management initiative that involved the joint formulation of comprehensive individual patient care plans.	Mixed method	Primary care	Canada	▪ Family physicians generally viewed chronic illness management from a predominantly biomedical perspective. Most found the strategy to be difficult to implement within existing organizational (time consuming) and financial constraints. Care planning conflicted with preexisting concepts of their role and of their patients abilities to become partners in care. The few patients who noticed the process spoke favorable about their experience. - This study found important individual-level barriers to chronic illness management in primary care. These issues seemed to transcend existing organizational and resource constraints. This study highlights the importance of personal attributes and perspectives of individuals in addition to larger system issues.
Stolee [56]	2013	Pubmed	This article presents a qualitative evaluation of the utility of Goal Attainment Scaling (GAS) in geriatric care.	Qualitative study	Nursing home	Canada	▪ Most felt that GAS did not affect the types of goals set for patients but was especially helpful in that the goals were more clearly specified and were more likely to relate to problems to which the program could actively respond (greater focus and direction).<br>▪ There seemed to be less likelihood of missing important things because of the multidisciplinary approach to goal setting.<br>▪ Interdisciplinary roles blurred. Helpful in encouraging greater emphasis on team goal-setting versus unidisciplinary goal setting.

							▪ GAS helped to make team conferences much shorter and more efficient.<br>▪ GAS encouraged greater connection, communication and cohesiveness among team members. Greater balance in goal setting
Trivedi [14]	2013	Expert panel	This review aimed to identify the models of interprofessional working that provide the strongest evidence base for practice with community dwelling older people.	Review	Primary care	U.K.	▪ A shared care plan is a shared document that involves joint input from an interprofessional team.<br>▪ The role of the case manager<br>▪ Interprofessional collaboration has the potential to improve outcomes, although studies are few and flawed with methodological limitations and mixed results.
Weklar [40]	1982	Pubmed	This article discusses the application of a team approach regarding patient care of elderly.	Discussion paper	Nursing home	U.S.A.	▪ A patient care management system is a systematic holistic approach to the care of the long- term patient. To ensure the comprehensiveness of the interdisciplinary assessment, all persons responsible for providing care, treatment and service to the patient need to participate. Once the assessment is made, an individualized patient-centered care plan can be formalized. The plan must be updated when changes occur. It is expected that clearly, delineated, time-limited goals will characterize the plan of care.<br>▪ In order to remain appropriate and responsive to the patient's current status, periodic evaluations are proposed which would review goal achievement and any changes in needs and thus new goals.<br>▪ The shared care plan has been described as a holistic approach which balances the emotional, social, physical and pathological needs.<br>▪ The coordinator is responsible for ensuring team members' participation, that the assessment is completed and that the care plan reflects the patients assessed needs.<br>▪ An administrator is responsible for ensuring the implementation of a patient care management system. Coordinating the various disciplines involved in the assessment team. Through his/her guidance, the interdisciplinary team can be firmly established within a facility. Create a suitable functional environment, coordinate interdepartmental and interdisciplinary activities and help form cohesive relationships
Wright [31]	1993	Pubmed	In this article there has been described how members of a multidisciplinary team applied their knowledge to formulate a care plan.	Observational study	Nursing home	U.S.A.	▪ Care plans generated in this study were handwritten at the time of the team conference and created as a result of team interaction.<br>▪ To create a plan of care, members of the team need an order, or format, to follow, a structure of interaction and common language.<br>▪ The group needs a leader and a recorder to document the goals and interventions.<br>▪ A plan is needed with action steps to reach the goal of a functioning multidisciplinary team. Within these actions the human element is the purpose.<br>▪ Goals set must be resident centered and achievable (realistic)

							▪ Implementing a care plan takes time and increased numbers of staff to perform the interventions noted on the plan of care.
Xyrichis [29]	2007	Expert panel	This review aimed to explore the factors that inhibit or facilitate interprofessional teamworking in primary and community care settings.	Review	Primary care	U.K.	▪ The terms multiprofessional, interdisciplinary and multidisciplinary are often used interchangeably in the literature.<br>▪ Functions of interprofessional healthcare teams working in the 21st century are complex, being influenced by many interrelating factors.<br>▪ Teamworking is recommended as a way of providing holistic care since team members skills, experience and knowledge are pooled together to produce the best outcome.<br>▪ Interprofessional working could achieve greater resource efficiency and improve standards of care through a reduction in duplication and gaps in service provision, enabling the delivery of holistic services.<br>▪ Two main themes emerged that had an impact on interprofessional teamworking: team structure and team processes. Within these two themes, six categories were identified: team premises; team size and composition; organizational support; team meetings; clear goals and objectives; and audit.<br>▪ The structure of the team emerged as a very important factor for effective teamworking.<br>▪ Smaller sized teams appear to function better than larger teams.<br>▪ Lack of clarity about leadership predicted lower levels of team effectiveness and was associated with poor quality teamworking.<br>▪ Organizational support both for teamworking and for the team's members is crucial to the effective working of a team.<br>▪ Regular team meetings were associated with effective teamwork.<br>▪ Teams fail to work effectively when explicit team goals are lacking.<br>▪ Training needs to be provided to enable healthcare professionals to gain the knowledge and skills required for effective teamwork.
Zawadski [42]	1988	Pubmed	This article describes the consolidated model of case management as used in On Lok's capitated long-term care program.	Discussion paper	Chronic care	U.S.A.	▪ Each professional individually assesses the participant according to a defined protocol. Upon completing the assessment of each case, a treatment plan is formulated by the team members, through a process of negotiation and agreement. Discussing the patients' needs and develop a coordinated service plan.<br>▪ Outcomes: reduction of high-cost hospital days. Reduction of nursing home days. Increase of community services.<br>▪ The consolidated model of case management is a combination of the assessment and monitoring functions with service coordination and delivery (the core is a framework of multidisc team management).<br>▪ The wishes and needs of the patient and / or family are incorporated into the assessment process and the treatment plan is discussed with and approved by them.

**The studies are presented in alphabetical order. The information is presented as found in the articles. The key findings of the included articles are presented in the last paragraph.. Key findings comprise relevant information about interventions, outcomes, barriers, facilitators, theories used, definitions and elements related to developing interprofessional shared care plans.*
